# Molecular epidemiology and phylogenetic analysis of Dengue virus type-1 and 2 isolated in Malaysia

**DOI:** 10.12669/pjms.313.6340

**Published:** 2015

**Authors:** Muhd Hasyim Chew, Md. Mostafizur Rahman, Salasawati Hussin

**Affiliations:** 1 Muhammad Hasyim Chew, Ph.D scholar, Laboratory Public Health Kota Kinabalu, Sabah, Ministry of Health, Malaysia; 2 Dr. Md. Mostafizur Rahman, Professor, Department of Medical Microbiology and Immunology, Universiti Kebangsaan Malaysia Medical Centre, Kuala Lumpur, Malaysia; 3 Dr. Salasawati Hussin, Associate Professor, Department of Medical Microbiology and Immunology, Universiti Kebangsaan Malaysia Medical Centre, Kuala Lumpur, Malaysia

**Keywords:** DENV, E gene, Genotype, Molecular epidemiology, Phylogenetic analysis

## Abstract

**Objective:**

Detection of different serotypes of dengue virus and provide information on origin, distribution and genotype of the virus.

**Methods:**

Dengue virus serotypes identified as DEN-1 and DEN-2 were amplified and sequenced with E gene. The consensus sequences were aligned with references E gene sequences of globally available GenBank. Phylogenetic analysis was performed using Neighbor-joining and Kimura 2-parameter model to construct phylogenetic tree.

**Results:**

A total of 53 dengue virus isolates were positive, of which 38 (71.7%) were DENV-1 and 15 (28.3%) were DENV-2. Phylogenetic tree of DENV-1 and DENV-2 showed that the isolates were clustered in genotype I and cosmopolitan genotype, respectively considered the predominant genotypes in Southeast Asian countries. The molecular epidemiology genotype I DENV-1 and cosmopolitan genotype DENV-2 have been co-circulating in Klang Valley areas, Malaysia without shifting of genotype.

**Conclusion:**

The study reveals that DENV-1 and DENV-2 have been circulating in Malaysia. The isolates are clustered in genotype 1 and cosmopolitian genotype, respectively. The study results would help in planning for prevention and control of dengue virus in Malaysia.

## INTRODUCTION

Dengue is the most prevalent arthropod- borne viral infection, transmitted by *Aedes. aegypti* caused major impact on health and economies in subtropical and tropical countries of the worldwide. The report of World Health Organisation (WHO) indicated that the incidence of dengue has dramaticaly increased 30-fold since 1955 to 2010 and estimated 50-100 million new infections occurred annually over 100 endemic countries especially hundreds of thousands of severe cases increased, in Southeast Asian countries.^1^,^2^

Dengue virus (DENV) is a member of the genus *Flavivirus*, family *Flaviviridae*. It is an envelope virus with length 11kb positive sense ssRNA genome. The genome is comprised of three main structural proteins, capsid (C), premembrane/membrane (prM/M) and envelope (E) and seven non-structural proteins; NS1, NS2a, NS2b, NS3, NS4a, NS4b and NS5. It causes different clinical manifestations ranging from a dengue fever (DF) to severe complications such as dengue hemorrhagic fever (DHF) and dengue shock syndrome (DSS).^3^,^4^ Infections with any of four serotypes would provide life-long protective homotypic immunity but not against the other three.^5^ Epidemiology and phylogenetic studies provide useful information concerning geographical distribution of genotype DENV in the different regions. Previous studies based on E/NS1 gene junction and complete E gene to measure genetic diversity in DENV-1 and DENV-2 revealed that sequence divergence was no more than 6%.^6^ The classification of genotypes based on the E gene has been described by authors Goncalvez et al.^7^, and Twiddy et al.^8^ for DENV-1 and DENV-2, respectively. Genetic studies of DENV isolates from patients or mosquitoes have made strong progress toward the understanding patterns of genotype abundance, replacements of new strain may lead to new epidemics and disease.^9^

Malaysia is a tropical country with hot and humid weather located in Southeast Asia between Thailand and Singapore. Dengue became one of the major public health problem when occured the largest outbreaks with over 6000 cases since 1991.^10^ To date, 43,346 dengue cases and 92 deaths were reported by Ministry of Health, Malaysia in 2013.^11^ It was shown that Malaysia is hyperendemic of dengue associated with 4 serotypes co-circulating in the country. The previous studies Vinomarlini et al.^12^, and Homes et al.^13^ revealed that co-circulating of 4 serotypes DENV in the study areas. The study also showed that DENV-3 was the highest case among the 4 serotypes followed by DENV-1, DEN-2 and DENV-4 during 2005-2009 in east coast of peninsular Malaysia.^12^ Teoh reported that DENV-1 genotype I and II causing major outbreak occurred in Klang valley in 1987, 1997 and 2004.^14^ Simultaneously, DENV-2 genotype cosmopolitan was a predominant in Malaysian strains in Asian mainly introduced from Thailand due to economic activities and travellers.^15^ However, the limited information on molecular epidemiology of DENV-1 and DENV-2 genotypes due to lack of existed document in Malaysia. Thus, this study was aimed to conduct providing information on origin, geographical distribuiton of DENV-1 and DENV-2 in Klang Valley areas.

## METHODS

### Ethical Approval

The research was approved by the ethical Committee of Universiti Kebangsaan Malaysia Medical Centre, Malaysia (FF247-2011).

### Viruses

A total of 313 acute-phase serum samples received from January 2011 to December 2012 in University Kebangsaan Malaysia Medical Centre (UKMMC), Kuala Lumpur, were used in this study. The 53 isolates of DENV-1 and DENV-2 were isolated from acute-phase serum samples. 20µl of serum was inoculated in C6/36 clone *Aedes Albopictus* (ATCC CRL-1660) monolayer cell and grown in Roswell Park Memorial Institute (RPMI) 1641media (Gibco, USA) with heat-inactivated 1% fetal calf serum (Gibco, USA). This was incubated at 30°C for 10d and then identified by Indirect Immunoflourescene assay (IFA) with polyclonal dengue complex antibody (MILIPORE, USA) and typed with 4 serotypes dengue specifics monoclonal antibody (MILIPORE, USA). The positive culture supernatant was recovered by centrifugation and stored at -80°C for further RT-PCR and sequencing test.

### Viral RNA extraction, RT-PCR and Sequencing

Viral RNA was extracted from 140 µl of virus infected culture fluid by using QIAamp Viral RNA kit (QIAGEN, Inc, Germany) according to manufacturer’s protocol. The serotype-specific primers envelope (E) gene initially used by Lee et al.^16^ to amplification and sequencing of genome DENV was applied in this study. The serotype-specific primers E gene listed in Table-I. The MyTaq One-step RT-PCR kit (Bioline, USA) was used to detect DENV-1 and DENV-2 according to manufacturer’s protocol. The 5ul of RNA was added to mixture of One-step RT-PCR kit and 10µM of reverse and forward serotype specific primers. The following profile protocol One-step RT-PCR amplifications step listed below:

**Table-I:** Serotype-specific primers for amplification and sequencing of partial E gene.

Serotypes	Primers	Sequence (5’- 3’)
DENV-1	D1-1229F	AGAGGCTGGGGCAATGG
	D1-1710R	GCTCCTTCTTGTGATCCTAGTAC
DENV-2	D2-1353F	GTGATAACACCTCACTCAGGG
	D2-1298R	CCTATAGATGTGAACACTCCTCC

**Table:** 

Steps	Temp.	Time	No. of cycle
Reverse transcriptase	50°C	30min	1
Initial PCR activation step	95°C	1min	1
Denaturation	95°C	10s	34
Annealing	50°C	10s	34
Extension	72°C	30s	34
Final extension	72°C	10min	1
Hold	4°C		

Gel electrophoresis was performed to verified PCR product size of serotypes DENV. Then, the PCR-amplified products were purified using Qiaquick PCR Purification Kit (QIAGEN, Germany) according to manufacturer’s protocol. Simultaneously, each purified PCR product was measured using NanoDrop 2000c (Thermo Scientific, USA) to ensure purity and concentration DNA are between range 1.8-2.0 and 20-25ng of eluted DNA, respectively. Purified product was outsourced to the 1st BASE company Malaysia for sequencing.

### Nucleotide, alignment and phylogenetic analyses

The 53 raw sequences partial E gene were analyzed, edited and BLAST search was performed using tblastx to confirm the similarity and identity of the sequences. Thus, the edited sequences were submitted to GenBank using Bankit for verification and registration sequences. The following detailed of accession numbers: KF030624 - KF030661 and KF030662 - KF030676 are shown in Table-II. The DENV isolates sequences were aligned together with various global reference sequences of different genotype available in GenBank with Clustal-X software.^17^ Phylogenetic analysis was performed using MEGA version 5.2.^18^ Phylogenetic tree was constructed by Neighbor-joining, Kimura 2-parameter method and bootstrap test with 1,000 replications. D2/NewGuinea/NGC/1944 strain (M29095) and D1/USA/Hawaii/1945 strain (AF425619), were used as out-group to root the tree.

**Table-II:** List of DENV-1 and DENV-2 isolated from Klang Valley areas during 2011-2012 were used for phylogenetic study.

Strains	Dengue serotypes	Year of isolate	Areas	GenBank Accession	Genotypes
MS12006453	1	2012	Cheras	KF030624	I
MS12006456	1	2012	Cheras	KF030625	I
MS11006520	1	2011	Cheras	KF030626	I
MS11006531	1	2011	Cheras	KF030627	I
MS12006563	1	2012	Cheras	KF030628	I
MS12006615	1	2012	Cheras	KF030629	I
MS11006630	1	2011	Cheras	KF030630	I
MS12006735	1	2012	Cheras	KF030631	I
MS11006821	1	2011	Cheras	KF030632	I
MS11006940	1	2011	Sungai Besi	KF030633	I
MS12006981	1	2012	Cheras	KF030634	I
MS11007000	1	2011	Ampang	KF030635	I
MS11007121	1	2011	Cheras	KF030636	I
MS12007135	1	2012	Cheras	KF030637	I
MS11007142	1	2011	Cheras	KF030638	I
MS12007357	1	2012	Cheras	KF030639	I
MS12007378	1	2012	Cheras	KF030640	I
MS11007747	1	2011	Cheras	KF030641	I
MS11008005	1	2011	Balakong	KF030642	I
MS12008160	1	2012	Cheras	KF030643	I
MS11008188	1	2011	Cheras	KF030644	I
MS12008384	1	2012	Cheras	KF030645	I
MS12008387	1	2012	Cheras	KF030646	I
MS11008684	1	2011	Sungai Besi	KF030647	I
MS12008824	1	2012	Cheras	KF030648	I
MS12009775	1	2012	Cheras	KF030649	I
MS11009783	1	2011	Kajang	KF030650	I
MS11009829	1	2011	Cheras	KF030651	I
MS11009883	1	2011	Cheras	KF030652	I
MS12010190	1	2012	Cheras	KF030653	I
MS11010383	1	2011	Cheras	KF030654	I
MS11010846	1	2011	Cheras	KF030655	I
MS11010993	1	2011	Cheras	KF030656	I
MS11011303	1	2011	Cheras	KF030657	I
MS11011622	1	2011	Cheras	KF030658	I
MS11011708	1	2011	Cheras	KF030659	I
MS11011709	1	2011	Cheras	KF030660	I
MS12012268	1	2012	Cheras	KF030661	I
MS12006405	2	2012	Cheras	KF030662	Cosmopolitan
MS11006707	2	2011	Cheras	KF030663	Cosmopolitan
MS12006891	2	2012	Cheras	KF030664	Cosmopolitan
MS12006899	2	2012	Cheras	KF030665	Cosmopolitan
MS12006909	2	2012	Cheras	KF030666	Cosmopolitan
MS11007164	2	2011	Sri Petaling	KF030667	Cosmopolitan
MS12007550	2	2012	Cheras	KF030668	Cosmopolitan
MS11008185	2	2011	Cheras	KF030669	Cosmopolitan
MS11008692	2	2011	Sungai Besi	KF030670	Cosmopolitan
MS11010075	2	2011	Puchong	KF030671	Cosmopolitan
MS11010358	2	2011	Seri Kembangan	KF030672	Cosmopolitan
MS11011115	2	2011	Cheras	KF030673	Cosmopolitan
MS11011149	2	2011	Cheras	KF030674	Cosmopolitan
MS11011405	2	2011	Cheras	KF030675	Cosmopolitan
MS11011411	2	2011	Sri Petaling	KF030676	Cosmopolitan

## RESULTS

Out of total 313 serum samples tested, 53(16.93%) were positive. Of 38 (71.7%) positive were DENV-1 and 15 (28.3%) DENV-2. The results revealed that DENV -1 and DENV -2 was co-circulating in various of areas of Klang Valley (Table-II).

### Phylogenetic analysis DENV-1 isolates

**Fig.1:**
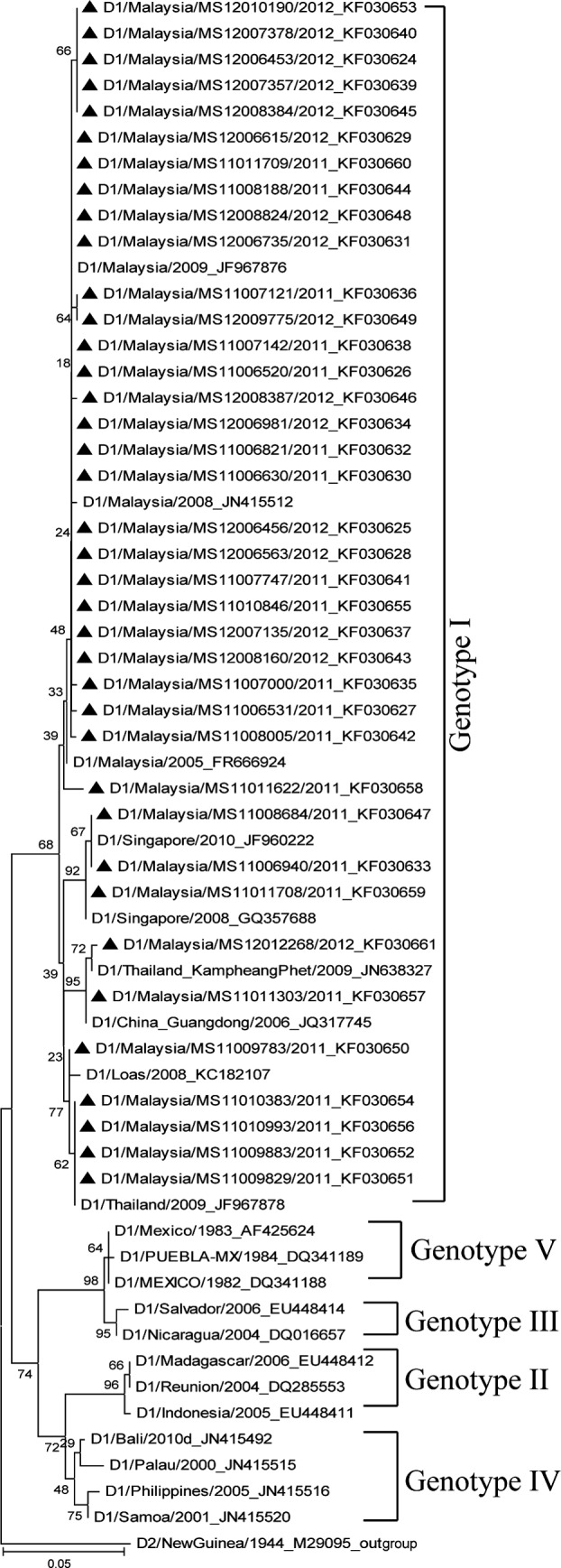
The phylogenetic tree of DENV-1 based on partial envelope (E) gene sequence isolates in Klang Valley 2011-2012. The tree was constructed by the neighbor-joining method (MEGA 5.2) with bootstrap 1000 replications. The 38 isolates DENV-1 were aligned with 21 reference sequences global from GenBank. The tree was rooted with out-group DENV-2 from New Guinea, accession number M29095. The Malaysian isolates are designated in black triangle and reference sequences were in abbreviations serotype/country/year of isolation and follow by GenBank accession number.

Of 38 DENV-1 sequences generated from the E gene were aligned with 21 reference strains of various genotypes. Fig.1 shows that the phylogenetic tree constructed by Neighbor-joining method reveals that all 38 isolates have been clustered into genotype I. Two clusters were identified in the tree from 28 isolates which originally known as local strain and 10 isolates from imported strains introduced from different geographical distribution. Of 28 DENV-1 isolates were closely related to the viral strains from Malaysia. In the study it reveals that 5 isolates were closely related to Thailand strains, 3 isolates closely related to Singapore strains, and 1 isolate closely related to China and Laos strain. It is noted that the imported dengue virus strains mostly originated from Southeast Asian region.

### Phylogenetic analysis DENV-2 isolates

**Fig.2:**
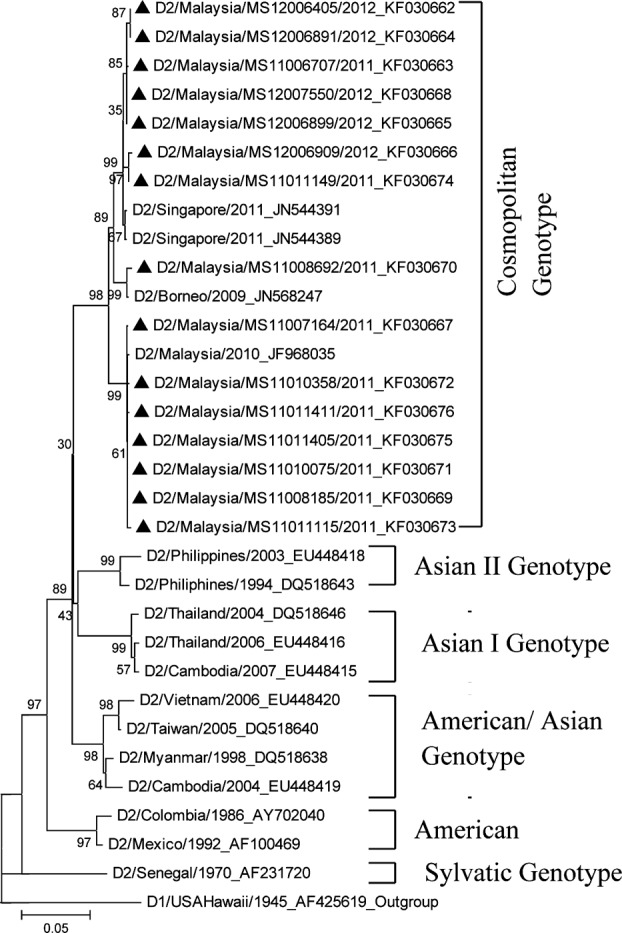
The phylogenetic tree of DENV-2 partial envelope (E) gene sequence isolates from Klang Valley 2011-2012. The tree was constructed by the neighbor-joining method (MEGA 5.2) with bootstrap 1000 replications. The 15 isolates DENV-2 were aligned with 15 references sequences global from GenBank. The tree was rooted with DENV-1 from Hawaii, USA as out-group, an accession number is AF231720. The Malaysia isolates designated in black triangle and reference sequences abbreviations in serotype/country/year of isolation and follow by GenBank accession number.

Fig.2 presents the sequences of 15 isolates DENV-2 and 16 reference strains of various genotypes from global available in Genbank were used in this phylogenetic analysis. The phylogenetic tree revealed that all the DENV-2 isolates were clustered into Cosmopolitan genotype. The cosmopolitan genotype contains viruses which similarity to Malaysia strains (n = 7), Singpore strains (n = 7) and Borneo strain (n = 1). The geographical distribution of DENV-2 in this study indicated that 7 isolates were originally from Malaysia and 8 isolates might were imported from neighbouring countries of Southeast Asia.

## DISCUSSION

The study was conducted for the understanding of genetic diversities and geographical distribution of dengue viruses in the Klang Valley in Malaysia. From these studies, Cheras was the highest infected dengue area followed by other areas Klang Valley (Table-I). Cheras has been declared as Hot Spot area by Ministry of Health (MOH) Malaysia in the year 2011 due to large number of outbreaks of dengue.^19^ In 2013, MOH reported 457 outbreaks occurred in Selangor and 19 of such outbreaks occurred in the Klang Valley.^11^ These outbreaks might have occurred due to mass transportation for global commercial activities and travelling industries that could lead to frequent exchange of dengue viruses from neighboring countries.

Phylogenetic analysis based on partial E gene sequence of 53 DENV-1 and DENV-2 isolates during 2011-2012 from Klang Valley provided genetic relationship of these viral strains. The determination of DENV genotypes are based on the classification of Goncalvez et al^7^, and Twiddy et al.^8^ From the present study of phylogenetic analysis DENV-1, revealed that 38 isolates clustered in genotype I and 28 isolates were found to be closely related to Malaysian strain and 10 isolates closely related to imported strains from Thailand (n = 5), Singapore (n = 3), Laos (n = 1) and China (n = 1). The recent study of Teoh et al.^14^ revealed that circulating of genotype I, II, and III of DENV-1 were associated with outbreaks in Klang Valley, Malaysia between year 2004, 1997 and 1987, respectively and occured replacement of genotypes from III to I due to importation of DENV-1 strain from the neighboring countries. Similarly our findings showed that 38 isolates clustered in genotype I in phylogenetic tree and these were predominant genotype circulating in that areas during 2011 to 2012. In a study Huang et al.^20^ revealed that DENV-1 genotype I in Vietnam and Thailand was the dominant genotype circurlating in Southeast Asia during 2008-2010, inducing viruses from neighboring countries. Others studies also indicated that DENV-1 genotype I had been circulated in Singapore^16^ (2008-2010), Laos^21^ (2007-2010), China^22^ (2001-2010) and Thailand^23^ (1992-2009). It is observed from the reports that the extensive introduction of new strains expedites local evolution of epidemic DENV strains in new areas could have made a dynamic change of clade and genotype shifting of dengue virus.

Fig.2 phylogenetic tree analysis of 15 isolates DENV-2 showed that 7 strains were closely related to Malaysia strains and others 8 strains found to be closely related to Singapore strains (n = 7) and Borneo strain (n = 1). These all isolates clustered under cosmopolitan genotype. Similar findings observed in previous studies which showed that cosmopolitan genotype was found in Klang valley (1989-2000) and Sarawak (1997-2002) and considered predominant genotype in Malaysia during the previous years. 13,15 It is showed that cosmopolitan genotype of DENV-2 strain was prevalent and maintained well in areas of study for the long time. It is proved that genotype distribution of DENV-2 remained stable in Malaysia for the last 23 years. The previous study^16^ also revealed that cosmopolitan genotype DENV-2 had been actively distributed in Singapore since 2000 and became predominant strain associated to the dengue outbreak during 2007-2009. This finding showed that Singapore strains had shared the same genotype of DENV-2 in Malaysia and exchanged DENV-2 strains between Malaysia and Singapore. Simultaneously the cosmopolitan genotype DENV-2 in Malaysia and Indonesian^24^ strains have been circulated to Asian countries like Taiwan and China^20^,^22^,^25^ as imported strains. The exchange of these virus strains would create more new outbreaks in the future.

Klang Valleys in Malaysia were the main areas of international trade and travel; place of migrant workers and site of construction buildings. This situation might have played the role for the exchange of virus strains due to human movement from abroad. It is assumed that DENV-1 and DENV-2 strains introduced into Malaysia mainly from Southeast Asian countries. The phylogenetic tree (Fig. 1 and 2) results showed that DENV-1 genotype 1 and DENV-2 cosmopolitan genotypes were co-circulating in the endemic areas, Klang Valley. DENV-1 genotype I and DENV-2 cosmopolitan genotype were predominant virus strains in this present study. The present study revealed that most of the dengue virus strains were imported from Singapore to Malaysia. As Singapore has become a destination for Malaysian for seeking work, education, and training opportunities which lead to frequent movement of population between these two countries, therefore it plays an important role for dengue dissemination.

Finally, the study showed that DENV-1 genotype I and DENV-2 cosmopolitan genotypes were distributed constantly in Klang Valley in Malaysia. The introduction of DENV strains to the new areas may create more outbreaks in the future. These results provide an upadated information of DENV strains and genotypes their geographical distributions and movement in the regions.
